# Epidemiology of spinal cord injury without radiographic abnormality in children: a nationwide perspective

**DOI:** 10.1007/s11832-016-0740-x

**Published:** 2016-05-21

**Authors:** Jeffrey Knox

**Affiliations:** Tripler Army Medical Center Orthopedic Surgery Service (MCHK-DSO), 1 Jarrett White Road, Honolulu, HI 96859 USA

**Keywords:** Sciwora, Trauma, Spinal cord injury, Epidemiology

## Abstract

**Purpose:**

To characterize the epidemiology and costs associated with spinal cord injury without radiographic abnormality (SCIWORA) based on patient age.

**Methods:**

An analysis of data complied for 2012 in the Healthcare Utilization Project KID database (HCUP-KID), which represents a nationwide database of pediatric admissions, was performed. An initial search identified all children diagnosed with SCIWORA based on International Classification of Diseases, 9th edition (ICD-9) codes. Only data on patients aged <18 years were included in the analysis. The associated codes were then searched to identify the cause of injury. Pertinent epidemiologic data were collected from the database, including age, gender, and racial group. Injury level and pattern were determined from the associated ICD-9 codes, as were associated injuries. Hospital data included length of stay, in-hospital mortality, total hospital charges, and primary payer. All data were compiled and stratified based on patient age into three groups: group 1, age 0–3 years; group 2, age 4–10 years; group 3, age 11–17 years. These data were compared using Student’s *t* test and Chi-squared analyses.

**Results:**

A total of 297 patients were identified who met the inclusion criteria. There was a slight predominance of females among the youngest patients (53 %) with a significant dominance of males in the oldest group (72 %) (*p* < 0.001). The most common race among the patients studied was white (50 %) followed by Hispanic (14 %), Black (12 %), Asian/Pacific Islander (4 %), and Native American (1 %). Overall, the most common cause of injury was sports injuries, which were responsible for 122/297 (41 %) injuries, followed by motor vehicle collisions (26 %). Mechanisms of injury were significantly varied based on age group, with motor vehicle collisions the most common cause in the youngest two age groups and sports injuries the most common in the oldest age group (*p* < 0.05). The most common location injured was the cervical spine (46 %), with the upper cervical spine most commonly injured, particularly in the younger age groups. Additional injuries were found in 158/297 (53 %) of patients, and these were more common among younger patients. Head trauma was the most common associated injury in all age groups, but the highest rate was found the youngest age groups (*p* < 0.0001). The average hospital stay for all patients was 13 days, with longer stays seen in younger age groups (*p* < 0.05). In-hospital mortality was uncommon among these patients and occurred in only 6/297 (2 %) of patients. Hospital charges were highest in the younger age groups, with an average charge of $210,772 for those in the youngest age group, decreasing to $72,178 for those in group 3 (*p* < 0.0005). The most common payer was public insurance/medicaid in the youngest age group and private insurance in groups 2 and 3 (*p* < 0.0001).

**Conclusions:**

SCIWORA is an uncommon but potentially devastating injury in children. As with many pediatric injuries, this injury is heterogeneous between children of differing ages. This analysis of a nationwide series of children with such injuries identified significant differences in injury location, causes of injury, associated injuries, and hospital charges associated with this diagnosis.

## Introduction

Spinal cord injury without radiologic abnormality (SCIWORA) was originally described by Pang and Wilberger in 1982 as a traumatic myelopathy without evidence of injury on radiographs or tomograms [1]. The injury is almost exclusively seen in children and is thought to occur due to the increased elasticity of the pediatric vertebral column that allows it to stretch past the tolerance of the spinal cord. The incidence of this injury varies significantly based on evidence from imaging studies and the definition used and ranges from 13 to 42 % of all pediatric spinal cord injuries [2–5]. As in other types of pediatric spinal cord injury, patient age is a key factor in this injury pattern. As the spine matures and develops, numerous anatomic and biomechanical changes occur which result in differences in injury frequency, pattern, and location [6–8]. Prior studies have shown SCIWORA to be a more common entity in younger children in whom it also occurs more frequently in the upper cervical spine compared to similar injuries in older children. While other important differences are likely present, few large-scale studies are available on this condition in the literature to further establish the key epidemiologic factors and the effect that age has on them. The aim of this study was to better characterize the epidemiology and available hospital data in a nationwide sample of children diagnosed with SCIWORA.

## Materials and methods

An analysis was performed utilizing the HCUP-KID database, which represents a nationwide sample of inpatient pediatric admissions. The 2012 dataset was queried as this dataset represents the most recent available data available. A search was performed to identify all children diagnosed with SCIWORA utilizing the International Classification of Diseases, 9th edition (ICD-9) codes (952.XX). Only data on patients aged <18 years were eligible for analysis. The associated external cause codes (E-codes) were searched to identify the cause of injury, and those patients without an identifiable traumatic etiology were excluded from analysis. The search included the identification of associated injuries to identify patients with an associated spinal fracture, dislocation, disc herniation, or other external cause of spinal cord injury. Patients with such diagnoses were also excluded from the analysis.

Pertinent data were collected from the database. Epidemiologic data included patient age and gender. ICD-9 codes were used to identify the level and pattern of SCIWORA, as well as to identify any injuries in addition to SCIWORA sustained. Minor associated injuries, such as contusions, superficial wounds, or abrasions, were not included in the analysis. Associated injuries were categorized into six groups: head injury (including injuries to the brain and skull); thoracic injuries (including rib fractures and pulmonary injuries); gastrointestinal injuries; orthopedic injuries (including fractures, tendon lacerations, and traumatic amputations); facial injuries; vascular injuries (including injuries to the peripheral or great vessels). Details of the hospital stay were collected, including length of stay, in-hospital mortality, and the occurrence of a major procedure. Procedures performed during the hospital stay were identified based on their ICD-9 code. Patients were divided into three groups based on age: group 1, age 0–3 years; group 2, age 4–10 years; group 3, age 11–17 years. This classification into these age groups has been previously shown to represent unique groups in terms of spinal trauma with significant between-group differences. To evaluate the pertinent differences between these age groups with regards to SCIWORA, pertinent data were then compared between these groups using Student’s *t* test and Chi-squared analyses.

## Results

A total of 297 patients were identified who met the inclusion criteria. Of these 297 patients, 53 were between 0 and 3 years of age and included in group 1 for analysis; 47 were between 4 and 10 years of age and included in group 2; 197 were between 11 and 17 years of age and included in group 3. There was a predominance of males in the entire patient population (191/297; 64.3 %). This male predominance varied between the three age groups, increasing with increasing age, with male patients accounting for 25/53 (47 %) patients in group 1, 24/47 (51 %) patients in group 2, and 142/197 (72 %) patients in group 3 (*p* < 0.0001).

### Causes of injury

Overall, the most common cause of injury was sports injuries which were responsible for 122/297 injuries (41 %), followed by motor vehicle collisions as the second most common cause of injury (76/297; 26 %), then by falls (14 %), assault (4 %), and being struck by a falling object (3 %). The injury mechanisms of 23 patients’ (8 %) were listed as “other,” and those of 11 (4 %) patients were unknown.

Mechanisms of injury varied significantly according to age group. In the youngest age group (group 1), motor vehicle collisions were the most common cause of injury, being responsible for 38 % of injuries. Falls were the second most common cause (23 %), followed by assault (17 %), and being struck by a falling object (8 %). No injuries were caused by sports in this group. In group 2, motor vehicle collisions remained the top cause (40 %) of injury, but sports injuries became the second most common cause (21 %), followed by falls (19 %) and being struck by a falling object (6 %). No injuries were caused by assault in this group. In the oldest age group (group 3), sports injuries were the most common cause of injury (112/297; 57 %), followed by motor vehicle collisions as the second most common cause (37/297; 19 %), then by falls (11 %), being struck by a falling object (2 %), and assault (1 %). The differences between all injury types were statistically significant (*p* < 0.05). Details on the injury types are given in Table 1.

**Table 1:** Causes of injury

Cause of injury	Group 1 (age 0–3 years;* n* = 53)	Group 2 (age 4 –10 years;* n* = 47)	Group 3 (age 11–17 years;* n* = 197)	*p* value
Motor vehicle collision	20 (38 %)	19 (40 %)	37 (19 %)	0.001
Falls	12 (23 %)	9 (19 %)	21 (11 %)	<0.05
Struck by a moving object	4 (8 %)	3 (6 %)	3 (2 %)	<0.05
Assault	9 (17 %)	0 (0 %)	2 (1 %)	<0.0001
Sports	0 (0 %)	10 (21 %)	112 (57 %)	<0.0001
Other	3 (6 %)	3 (6 %)	17 (9 %)	NS
Unknown	3 (6 %)	3 (6 %)	5 (3 %)	NS

Data are presented as the number (of patients) with the percentage given in parenthesis

NS, Not significant

### Injury location

The cervical spine was the most common location of injury in this study population, with cervical spine injuries present in 137/297 patients (46 %). The most common cervical spine injuries were upper cervical injuries, located between C1 and C4, and injuries in this location were present in 91/297 (31 %) of patients. Lower cervical injuries were much less common and only present in 19 % of patients. The thoracic spine was the second most common location of injuries, and 41 of the 297 patients (14 %) had injuries at this location. There was an equal distribution between upper and lower thoracic injuries (7 % each). Injuries to the lumbar spine were much less common and only seen in 21/297 (7 %) patients. Only one case of sacral SCIWORA was reported. Nine patients were reported to have multiple level SCIWORA, but the specific locations of the injury were not reported. SCIWORA of an unspecified location was reported in 101/297 (34 %) patients.

Significant differences in injury location were identified between the different age groups. Cervical spine injuries were more commonly seen in the youngest age group, being present in 64 % of patients. Cervical injuries became less common in both groups 2 and 3 (53 and 40 %, respectively) (*p* < 0.05). Similarly, upper cervical spine injuries were most common in the youngest age group (51 %) and progressively less common in groups 2 and 3 (40 and 23 %, respectively) (*p* < 0.0001). The rates of lower cervical spine, thoracic, lumbar, and sacral injuries did not show any statistically significant differences between the age groups. SCIWORA of an unspecified type was most commonly seen in the oldest age group (43 %), becoming progressively less common in groups 2 and 1 (28 and 8 %, respectively) (*p* < 0.0001). Details on the locations of injuries are given in Table 2.

**Table 2:** Locations of injury

Location of injury	Group 1	Group 2	Group 3	*p* value
Cervical	34 (64 %)	25 (53 %)	78 (40 %)	<0.05
Upper cervical	27 (51 %)	19 (40 %)	45 (23 %)	<0.0001
Lower cervical	9 (17 %)	6 (13 %)	40 (20 %)	<0.05
Thoracic	11 (21 %)	4 (9 %)	26 (13 %)	0.190
Upper thoracic	5 (9 %)	1 (2 %)	14 (7 %)	0.325
Lower thoracic	6 (11 %)	3 (6 %)	13 (7 %)	0.759
Lumbar	7 (13 %)	3 (6 %)	11 (6 %)	0.155
Sacral	0 (0 %)	0 (0 %)	1 (1 %)	NS
Multiple	3 (6 %)	2 (4 %)	4 (2 %)	NS
Unspecified	4 (8 %)	13 (28 %)	84 (43 %)	<0.0001

Data are presented as the number (of patients) with the percentage given in parenthesis

For details on each age group, see Table 1

### Associated injuries

The SCIWORA syndrome represents a high-energy injury which is commonly associated with additional injuries. Concomitant injuries were found in 158/297 (53 %) patients in this series and were progressively more common in the younger age groups. Of the 53 patients in group 1, 46 (87 %) sustained a total of 75 additional injuries, yielding an average of 1.6 injuries per patient. Of the 47 patients in group 2, 31 (66 %) sustained a total of 43 additional injuries, yielding an average of 0.9 injuries per person. Finally, in group 3, 81/197 (41 %) patients sustained a total of 95 additional injuries, yielding an average of 0.5 injuries per person (*p* < 0.0001). Details on concomitant injuries are given in Table 3.

**Table 3:** Concomitant injuries

Concomitant injury	Group 1	Group 2	Group 3	*p* value
All injuries	46 (87 %)	31 (66 %)	81 (41 %)	<0.0001
Brain/head trauma	34 (64 %)	22 (47 %)	56 (28 %)	<0.0001
Thoracic	10 (19 %)	7 (15 %)	11 (6 %)	0.005
Gastrointestinal	7 (13 %)	4 (9 %)	1 (1 %)	<0.0001
Orthopedic	12 (23 %)	6 (13 %)	13 (7 %)	0.003
Facial	12 (23 %)	4 (9 %)	12 (6 %)	0.001
Vascular	0 (0 %)	0 (0 %)	2 (1 %)	0.6

Data are presented as the number (of patients) with the percentage given in parenthesis

For details on each age group, see Table 1

While head trauma was the most common injury among all age groups, such injuries were most common among the youngest patients, with 34/53 patients (64 %) in the youngest age group (group 1) sustaining head trauma, compared to 47 % of those in group 2 and 28 % of those in group 3 (*p* < 0.0001).

Orthopedic injuries were the second most common injuries overall and were present in 31/297 (10 %) of patients. Orthopedic injuries were more common among the youngest patients, being present in 23 % of patients in group 1 as compared to 6/47 patients (13 %) in group 2 and only 13/197 (7 %) patients in group 3. These between-group differences were statistically significant (*p* = 0.003).

Facial injuries were found in 28/297 (9 %) of patients overall. These type of injuries were more commonly seen in the youngest patients, being present in 12/53 (23 %) of patients in group 1 as compared to 4/47 patients (9 %) in group 2 and 12/197 (6 %) in group 3. These between-group differences were statistically significant (*p* = 0.001).

Thoracic injuries were seen in 28/297 (9 %) of patients overall. These injuries were more commonly seen in the youngest patients and were present in 10/53 (19 %) of patients in group 1 as compared to 7/47 (15 %) of patients in group 2 and only 11/197 (6 %) of patients in group 3. These between-group differences were statistically significant (*p* = 0.005).

Gastrointestinal injuries were rare in this patient population and were only seen in 12/297 patients overall (4 %). Such injuries were also more commonly seen in the younger patients, with 7/53 (13 %) of patients in group 1 demonstrating such injuries as compared to 4/47 (9 %) of patients in group 2 and only ½97 (1 %) in group 3. These between-group differences were statistically significant (*p* < 0.0001).

Only two patients in the patient population analyzed were found to have vascular injuries in this study; both patients were in group 3.

### **Hospital course**

The average hospital stay for all patients was 13 days. Younger age was associated with longer hospital stays. Patients in group 1 had an average length of hospital stay of 20.2 days, compared to 12.5 days for patients in group 2 and 5 days for those in group 3. These differences were statistically significant with the exception of group 1 versus group 2.

Of the 297 patients, 56 (19 %) underwent a total of 122 major procedures during the hospital stay. Major procedures were performed more commonly in patients in the younger age groups. In group 1, 21/53 (40 %) patients underwent a total of 25 procedures, with intracranial procedures being the most common type of procedure performed in this age group (15 % of patients). The second most common procedure for the youngest patients were spinal procedures (13 %), following by gastrointestinal (11 %), orthopedic (6 %), and facial (2 %) procedures. In group 2, 13/47 (28 %) patients underwent a total of 15 procedures during their hospital stay, with the most common procedures in this group being spinal procedures (13 %), followed by gastrointestinal (9 %), intracranial (6 %), and orthopedic (4 %) procedures. In group 3, only 22/197 patients underwent a total of 29 procedures. Spinal procedures were the most common in this group (6 %), followed by intracranial, gastrointestinal, vascular, and orthopedic (2 % each) procedures, and facial, genitourinary, and thoracic procedures (1 % each).

In-hospital mortality was uncommon among the 297 patients, with only six (2 %) patients dying during the hospital stay. The highest mortality rate was seen among patients in group 2 (4 % mortality rate), followed by those in group 3 (2 % mortality rate). None of the patients in group 1 died.

## Discussion

Spinal cord injury without radiographic abnormality (SCIWORA) represents a relatively uncommon but potentially devastating injury among children. Here, I have evaluated a nationwide sample of children with this diagnosis to better understand the epidemiology and costs associated with this injury and significant differences found between different age groups. It should be noted that the relative rate of SCIWORA versus other types of spinal injuries was not evaluated in the present analysis as information on this subject is available in the literature. Piatt recently evaluated a similar patient sample and found SCIWORA to represent nearly 20 % of injuries in children aged less than 3 years, 9.4 % of injuries in children aged 3–12 years, and only 5 % of injuries in patients aged 13–20 years [9].

The patient series in the present analysis demonstrated a significant variability in injury mechanisms that was based on age. This result is similar to those reported in prior studies evaluating all types of spinal injuries which showed higher rates of falls in younger patients, with sports injuries become predominant in older patients. In the series by Ossenbach and Menezes, birth trauma was a common cause, resulting in 33 % of injuries; however, this finding has not been shown in other series [5]. While birth trauma was not identified as a cause of SCIWORA in the present patient series, a significant number of patients were found to have sustained SCIWORA from non-accidental trauma. This was the third most common cause of injuries in the youngest age group and responsible for 17 % of cases in that group. Non-accidental trauma is an often under-recognized cause of spinal trauma in very young children; however, recent reports have highlighted the importance of this as a cause of other types of spinal injuries in the very young [10, 11].

Another important factor related to patient age is the location of the injury. Prior reports have demonstrated that SCIWORA in younger children occurs more commonly in the upper cervical spine, while SCIWORA in adolescents is more often located in the lower cervical and thoracic spine [1, 12–15]. The patients in the present series showed significant variability in injury location based on age, with higher rates of upper cervical spine injuries in younger children and higher rates of lower cervical spine injuries in older children. No significant difference was found between the rates of thoracic and lumbar injuries. However, a statistically significant increase in unspecified level SCIWORA in older children was identified which may represent a confounding factor in the data.

Injury severity is also significantly affected by patient age, with younger children more often sustaining complete and severe neurologic injuries. Dickman et al. found that 70 % of injuries in young children are neurologically complete at the time of presentation [16]. Pang et al. showed that 21/30 children under 8 years of age sustained complete or severe injuries compared to just ½5 children older than 8 [15] years. Injury severity is a key factor in these patients as it is strongly linked to the overall outcome of the injury [15, 17]. Patients with mild to moderate injuries have a good potential for functional recovery, with a potential for regaining full function [2, 17]. Hamilton and Myles evaluated the recovery potential of incomplete injuries and demonstrated that 74 % of patients showed significant improvement, with 59 % experiencing a complete recovery of neurologic function [2]. The prognosis for complete injuries, however, was much less promising, with <10 % of such injuries showing significant recovery [16].

It is important for the clinician to recognize that SCIWORA often represents high-energy trauma with a high rate of concomitant non-spinal injuries. The rate and nature of such injuries in the present patient series varied significantly based on patient age, with the youngest patients experiencing the highest rate of additional injuries, particularly injuries to the head, face, and extremities. Recognition of such differences is important in evaluating these challenging patients.

A significant difference in length of hospital stay was identified among the children with SCIWORA according to age, with a 2.6-fold longer stay among young children compared to the oldest patients. The reason behind the increased hospital stay is not clear and is likely multifactorial. The younger children demonstrated higher rates of additional injuries and higher rates of head trauma and also had higher rates of upper cervical level injury. As such, this result highlights the significant burden to the healthcare system inherent to such complex injuries.

In conclusion, the analysis reported here identified a large number of patients sustaining SCIWORA injuries in a nationwide database sample. Multiple significant factors were identified in these children that differ based on patient age. This finding highlights the complex nature of pediatric spinal trauma as well as the importance of differentiating between such age groups in research on pediatric spinal conditions.
